# The PAPER Study (Prescribing Antidepressants in Primary care: Ethnic inequalities in tReatment): a study protocol

**DOI:** 10.3399/BJGPO.2024.0311

**Published:** 2025-09-10

**Authors:** Lydia Poole, Amy Ronaldson, Hannah Frith, Paramjit Gill, Madiha Sajid, Rose Rickford, Andrea Martinez, Khaula Ali, Mel Ramasawmy

**Affiliations:** 1 Department of Psychological Interventions, University of Surrey, Guildford, UK; 2 Department of Health Service & Population Research, Institute of Psychiatry, Psychology & Neuroscience (IoPPN), King’s College London, London, UK; 3 Warwick Applied Health, Warwick Medical School, University of Warwick, Coventry, UK; 4 PPI representative, Coventry, UK; 5 College Road GP Practice, Woking, UK; 6 Wolfson Institute of Population Health, Queen Mary University of London, London, UK

**Keywords:** depression, primary health care, South Asian people, antidepressants, ethnic and racial minorities, UK Biobank, qualitative research

## Abstract

**Background:**

South Asian people represent the largest minority ethnic group in the UK, but prior research has suggested unequal access to primary care and differences in antidepressant prescribing practices for these patients.

**Aim:**

To understand the treatment of depression in South Asian patients, with specific reference to factors affecting appropriate prescribing. The secondary aim is to understand the intersection between ethnicity, age, and financial deprivation within this context.

**Design & setting:**

A mixed-methods approach will be adopted, including primary and secondary analyses, to understand the ways in which inequalities may arise along the pathway from patient experience of symptoms to clinician decision to treat with antidepressants in UK primary care.

**Method:**

Two scoping reviews will inform our approach. Quantitative data analysis of UK Biobank will allow us to examine prevalence and heterogeneity in depressive symptoms, and antidepressant prescribing over time, stratified by ethnicity. Qualitative data will be generated through interviews and focus group discussions with patients and healthcare professionals to understand experiences of depression and document the depression management decision-making process.

**Conclusion:**

The PAPER study will produce clinically relevant findings to support the treatment and management of depression in primary care for South Asian patients. The dissemination plan will be informed by patient and public involvement (PPI) group members and engagement with stakeholders. Our main outputs will include a toolkit of resources for use in primary care as well as community-facing materials.

## How this fits in

The symptom presentation of depression in South Asian people in the UK is thought to differ to that of other ethnic groups but has yet to be systematically studied. Barriers to help-seeking for depression for South Asian people in the UK include stigma, institutional racism, and cultural dissonance. As a result, appropriate prescribing of antidepressants for South Asian patients accessing primary care services can be challenging. The PAPER study seeks to produce up-to-date, practice-relevant findings to inform the diagnosis and management of depression in primary care for South Asian patients.

## Introduction

An estimated 300 million people worldwide are thought to live with depression, and major depressive disorder is recognised as a leading cause of years lived with disability globally.^1^ South Asian people comprise the largest minority ethnic group in the UK;^2^ however, data regarding the prevalence of depression by minority ethnic status relative to the White majority population is mixed.^3,4^ Research suggests that depression screening tools are less reliable at detecting cases of depression among British South Asian people, possibly owing to differences in preferred language and/or diction for emotions.^5^ The use of depression screening tools can precede the prescription of antidepressants in primary care, and higher severity scores are associated with higher prescribing and onward referral rates.^6^ While translated versions of standardised screening tools exist,^7,8^ these approaches focus on item-by-item translation, without consideration of cross-cultural differences in symptom perception and reporting. As acknowledged by this latter study, *‘instruments and diagnostic criteria may need to be adapted for use in primary care*’.^8^

Differences in depressive symptom experiences by ethnicity have been reported; notably lower recording of symptoms of guilt, hopelessness, and suicidal ideation have been observed in British South Asian patients.^9^ A review of qualitative studies looking at differences in depression symptoms across the world found the most prevalent symptoms reported in South Asian countries were related to somatic experiences.^10^ It is not clear to what extent acculturation may affect symptom heterogeneity by ethnicity; however, differences in presentation in primary care could contribute to diagnosis and treatment inequalities by ethnicity. Moreover, healthcare professionals’ own attitudes can act to compound the complex decision-making process around depression management, including beliefs that a diagnosis may be stigmatising for some patients.^11^

The National Institute for Health and Care Excellence (NICE) emphasise patient choice in treatment for less severe and severe new episodes of depression, one option being antidepressant medication.^12^ Antidepressant prescribing is often a first-line treatment for depression, despite efforts to increase access to non-pharmacological treatment.^13^ Rates of antidepressant prescriptions consistently increase year-on-year in the UK.^14^ Qualitative research has shown that GPs prefer to take the approach of ‘wait and see’ but if symptoms are perceived to be persistent, unresolving, severe, and ‘classic’, antidepressants will be prescribed earlier.^15^ Fear of depression recurrence, alongside low levels of concern regarding medication safety, and lack of proactive medication review (for example, patients only present in crisis), are all thought to drive antidepressant prescribing growth over time.^16^

Treatment of depression intersects with ethnicity. Social stigma can impede help-seeking^17^ and institutional racism and cultural dissonance may marginalise South Asian communities from access to mental health care.^18^ Data from a 2008 study found that Pakistani women living in the UK consulted with their GP more frequently than their White counterparts but were less likely to receive depression treatment.^19^ Language, feelings of isolation, and lack of adherence to mental health treatment regimens, which are often seen as unnecessary, can inhibit treatment.^20^ Given the upward trend in antidepressant prescribing overall, the lack of recent data considering the post-COVID-19 milieu, and the lack of data from individual South Asian groups, further research is needed.

### Aims and objectives

This study aims to understand the treatment of depression in South Asian patients, with specific reference to factors affecting appropriate prescribing in this patient group. A secondary aim is to understand the intersection between ethnicity, age, and deprivation within this context.

The objectives are as follows:

1. to estimate the prevalence of depressive symptoms and antidepressant prescribing rates among minority ethnic individuals in the UK;
2. to understand the presentation of depression in South Asian people and if existing depression screening tools used in primary care are culturally competent;
3. to identify aspects of the GP–patient consultation that affect antidepressant prescribing at the point of diagnosis or first-ever antidepressant prescription among South Asian patients; and
4. to use mixed methods to synthesise results across the work packages (WPs) to co-produce practice-relevant resources aimed at improving treatment of depression for South Asian people in primary care.

## Method

### Study design

**Figure 1.**
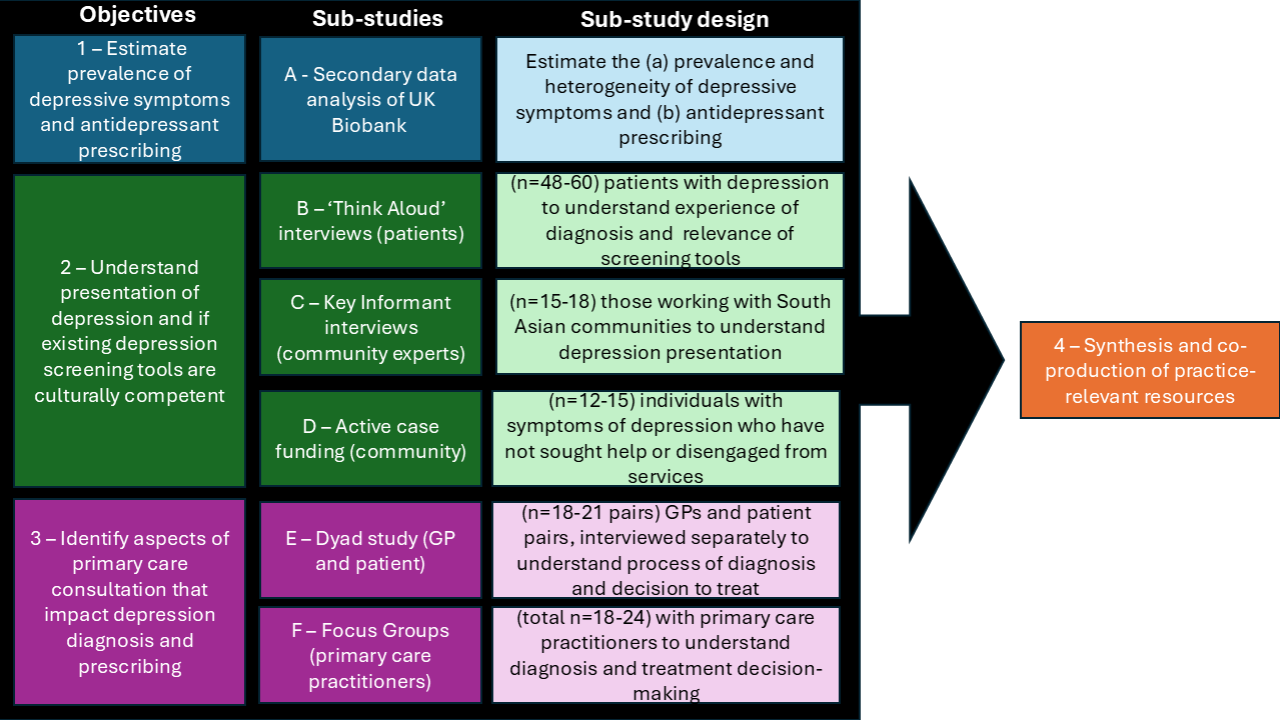
An overview of the PAPER Study objectives, sub-studies, and sub-study research designs

We will combine quantitative and qualitative approaches, analysing both primary and secondary data. Two scoping reviews of the existing literature will shape our approaches and aid the interpretation of findings (protocols: https://doi.org/10.17605/OSF.IO/5E6ZK and https://doi.org/10.17605/OSF.IO/EBHWX). We will have six empirical sub-studies, to capture a range of voices, experiences, and perspectives across the domains of the conceptual framework of access to health care.^21^ An overview of these sub-studies is set out in Figure 1.

### Quantitative methods and analysis

Quantitative methods will be used to examine trends across minority ethnic groups to enable us to examine differences in depression reporting and prescribing.

- **UK Biobank study**. We will conduct secondary analyses of the UK Biobank cohort^22^ to estimate the (a) prevalence and heterogeneity of depressive symptoms and (b) antidepressant prescribing among minority ethnic individuals in the UK. These analyses are important so that we can better understand the extent to which South Asians are under or over-represented in the data, as well as any differences by intersecting measures of inequality such as age, sex, socioeconomic position, and country of birth. A comprehensive data analysis plan is available (https://doi.org/10.17605/OSF.IO/EZR6J).

### Qualitative methods

Qualitative methods will be used to understand patient and public experiences of help-seeking and management of depression in primary care. Online or face-to-face interviews and focus groups will be the preferred method for data collection but telephone interviews will be made available where required. Access to an interpreter will also be available. The following sub-studies will be conducted:

- **‘Think-aloud’ study**. Patients with a recent or current diagnosis of depression will be recruited from primary care to participate in interviews (*n* = 48–60). Participants will be invited to share their experience of depression symptoms and help-seeking. The think-aloud method^23^ will be used to prompt participants to reflect on the relevance of either the Patient Health Questionnaire-9 (PHQ-9) or Hospital Anxiety and Depression Scale (HADS) questionnaire items to their own experiences of living with depression. This will allow us to make recommendations for how these screening tools can be improved and identify any symptoms that they do not adequately address.
- **Key informant study**. We will recruit (*n* = 15–18) individuals who work with South Asian communities to represent a range of perspectives in understanding depression presentation, via community and professional networks. This may include charities, community organisations, religious leaders, local healthcare commissioners, and health and care practitioners. The constant comparison method will be used to adapt recruitment and suggest additional groups or viewpoints as findings emerge and gaps are identified, such as including the views of informal caregivers. Interviews will seek to understand the discourses around depression and mental illness, cultural preferences in help-seeking and challenges in communicating symptoms of depression that may influence the diagnostic process of depression in primary care.
- **Community case-finding study**. We will engage with community organisations which will be briefed to identify individuals (*n* = 12–15) from a South Asian background who may experience symptoms of depression but have not sought help or advice, or who have made past attempts to seek help but subsequently disengaged, from primary care or other NHS services. Participants will be invited to interviews (conducted with an interpreter if necessary to allow them to speak in their preferred languages) to discuss their experiences, management approaches, and barriers to access to primary care.
- **Primary care dyad study**. We will invite GPs (*n* = 15–21) and the corresponding South Asian patients (*n* = 15–21) to interview. We will use the critical incident technique^24,25^ to ask them to relive the consultation in which the patient was diagnosed with depression, and a decision for management was taken (for example, prescribing, social prescribing, talking therapies, lifestyle management). Interviews will be conducted with healthcare professionals and patients separately, to ensure that both groups feel comfortable to share their views and to maintain confidentiality. To reduce recall bias, we will concentrate on consultations that took place in the previous month. This study will allow us to explore issues around presentation of depression symptoms, chronicity and severity of presenting symptoms, treatment advice sought and provided, communication challenges, and stigma.
- **Primary care focus group study**. We will conduct online focus groups with primary care practitioners responsible for diagnosis and prescribing for depression (*n* = 18–24; three groups of 6–8 participants in each). We will present patient case studies to understand the factors affecting antidepressant prescribing practices for a variety of depression presentations. We will use these focus groups to explore decision-making processes related to uncertainty, prescribing, onward referral, exploration of depression history and prior trauma and issues around language, cultural taboos, and health literacy.

### Qualitative data analysis

For the qualitative studies, audio-recordings will be made of all interviews and focus group discussions and will be transcribed verbatim. Data will be de-identified prior to analysis. Data will be analysed using reflexive thematic analysis^26^ in order to capture the experiences between and within participants. We will draw on the conceptual framework of access to health care^21^ to shape understanding of factors impacting access and outcomes of help-seeking for depression. We will use NVivo (version 14) to aid analysis.

### Synthesis, stakeholder engagement, and dissemination

We will draw together the findings from across the sub-studies, mapping our results onto the conceptual framework of access to health care,^21^ which we will employ as a conceptual guide to the integration of our findings. We will test our findings in knowledge exchange events with patients, healthcare professionals, and other key stakeholders to inform production of study outputs, including a toolkit for primary care and patient-facing materials.

### Patient and public involvement

Patient and public involvement (PPI) representatives sit on both the research team and steering committee. The PPI members have informed the PPI strategy and been involved in the study design and the development of written and verbal communication for participants. PPI will be involved in the interpretation of findings and development of policy recommendations.

## Discussion

Ethnic inequalities in health represent a matter of ongoing public enquiry in the UK.^27^ It is therefore imperative to understand access and delivery of care from the perspective of patients, healthcare professionals, and stakeholders in minority ethnic mental health care. Moreover, the *NHS Long Term Plan* ^28^ brings to the fore the need for reform for both adult mental health care for common mental illnesses (such as depression) as well as health inequalities (including those by ethnicity).

The PAPER Study has a number of limitations that we will attempt to minimise. UK Biobank is not a nationally representative cohort and therefore the generalisability of our findings will be reduced. We recognise that ‘South Asian’ represents a diverse group of ethnicities with differences in religion, language, and culture.^29^ Our sampling frame for our qualitative studies will, therefore, aim to recruit from the three largest South Asian diasporas in the UK (Indian, Pakistani, and Bangladeshi) while also attending to differences in generation, age, and sex. We also acknowledge that primary care practice varies between and within regions, making attempts to generalise our findings to the broader UK context challenging. We will address this through engaging with Regional Research Delivery Networks across the UK to promote a wide geographical reach for recruitment and use stakeholder events and PPI to inform the interpretation of our findings.

The PAPER Study will produce clinically relevant findings. Depression treatment involves collaborative decision making between primary care practitioners and their patients. It is not clear how such decision making takes place in the context of minority ethnic patients owing to differences in clinical signs (for example, heterogeneity in depression symptom presentation) and functional signs (for example, language, stigma, diction, mental health literacy, advocacy). Our work therefore offers the opportunity to shed light on these processes to make recommendations for best practice in primary care.

## Data

Data generated from the new qualitative studies will be publicly archived with participant consent following the end of the project.
